# Genomic Analysis of Hematopoietic Stem Cell at the Single-Cell Level: Optimization of Cell Fixation and Whole Genome Amplification (WGA) Protocol

**DOI:** 10.3390/ijms21197366

**Published:** 2020-10-06

**Authors:** Chiara Carretta, Selene Mallia, Elena Genovese, Sandra Parenti, Sebastiano Rontauroli, Elisa Bianchi, Sebastian Fantini, Stefano Sartini, Lara Tavernari, Enrico Tagliafico, Rossella Manfredini

**Affiliations:** 1 Centre for Regenerative Medicine “S. Ferrari”, University of Modena and Reggio Emilia, 41125 Modena, Italy; 187003@studenti.unimore.it (C.C.); selene.mallia@unimore.it (S.M.); 223191@studenti.unimore.it (E.G.); sandra.parenti@unimore.it (S.P.); sebastiano.rontauroli@unimore.it (S.R.); elisa.bianchi@unimore.it (E.B.); sebastian.fantini@unimore.it (S.F.); 268146@studenti.unimore.it (S.S.); lara.tavernari@unimore.it (L.T.); 2 Center for Genome Research, University of Modena and Reggio Emilia, 41125 Modena, Italy; enrico.tagliafico@unimore.it; 3 Department of Medical and Surgical Sciences, University of Modena and Reggio Emilia, 41125 Modena, Italy

**Keywords:** single-cell genomics, hematopoietic stem cells, myeloproliferative neoplasms, whole genome amplification, single-cell isolation, clonal architecture, clonal heterogeneity

## Abstract

Single-cell genomics has become the method of choice for the study of heterogeneous cell populations and represents an elective application in defining the architecture and clonal evolution in hematological neoplasms. Reconstructing the clonal evolution of a neoplastic population therefore represents the main way to understand more deeply the pathogenesis of the neoplasm, but it is also a potential tool to understand the evolution of the tumor population with respect to its response to therapy. Pre-analytical phase for single-cell genomics analysis is crucial to obtain a cell population suitable for single-cell sorting, and whole genome amplification is required to obtain the necessary amount of DNA from a single cell in order to proceed with sequencing. Here, we evaluated the impact of different methods of cellular immunostaining, fixation and whole genome amplification on the efficiency and yield of single-cell sequencing.

## 1. Introduction

The neoplastic evolution results from the accumulation of mutations and from the competition among subclones carrying different combinations of genetic lesions. 

It has been shown that the temporal order in which mutations accumulate in hematological neoplasms and in many solid human tumors, such as breast [1], kidney [2], colon [3] and prostate [4] cancers, is fundamental to understand the clonal landscape underlying disease evolution. In recent years, single-cell genomics has been developed as a powerful method for reconstructing the complex cancer pathogenesis [5] and its heterogeneity. The study of the mutational status of a tumor cell population at the single-cell level allowed to overcome some of the limitation of bulk sequencing such as being unable to discriminate the zygosity of mutations in each clone, thus not allowing to obtain a correct information about the clonal phylogeny [6].

The extraordinary perspectives offered by this technique are however counterbalanced by technical difficulties due to single-cell manipulation. Single-cell genomics approaches require two main critical steps that precede the downstream single-cell sequencing: single-cell isolation and single-cell whole genome amplification (scWGA). Several strategies can be used for the isolation of single cells, the most used of which is Fluorescence-activated cell sorting (FACS). This certainly has the advantage of not needing a cell fixation step. Nevertheless, it has two main drawbacks: the first is represented by the fact that the maximization of cellular recovery generally causes a decrease in cell purity. The DEPArray technology allows to couple high cell recovery while maintaining high levels of cell purity (purity = 100%). Furthermore, another advantage of the DEPArray compared to FACS sorting is that it automatically checks that only a single viable cell has been sorted, saving the researcher from manually checking for wells effectively containing only single cells.

Therefore, we adopted the DEPArray sorting even if it requires a cell fixation step. Cell fixation can introduce some bias during scWGA because of the effects exerted by fixative agents upon chromatin accessibility [7], resulting in the difficult amplification of genomic regions crosslinked to histone residues.

Several scWGA strategies have been developed in order to get a uniform and abundant amplification of single-cell genomic DNA. The main amplification methods available to date are based on the Multiple Displacement Amplification (MDA) [8], Degenerative Oligonucleotide PCR (DOP-PCR) [9] or Multiple Annealing and Looping-Based Amplification (MALBAC) [10]. These methods have been designed using different DNA fragmentation and amplification methods, some of them are PCR-based while others use an isothermal amplification system. As a result of amplification, these whole genome amplification (WGA) methods can exhibit different degrees of Allelic Dropout (ADO), which can be limiting for the downstream steps. ADO is the loss of one allele during PCR amplification and is an insidious pitfall introducing false homozygosity and therefore genotyping errors.

Myeloproliferative Neoplasms (MPNs) are a complex disease model due to their heterogeneous genetic landscape. The different mutational assets harbored by the patients determine their pathogenic evolution and response to treatment [11]. In hematopoietic malignancies, hematopoietic stem cells (HSCs) originate the disease and the cellular clones responsible for the pathologic onset and progression. Here, we demonstrate that the optimization of the pre-analytical phase is crucial to allow an efficient single-cell isolation and WGA. In particular, two different cell fixation methods were tested along with five commercially available WGA kits to obtain single-cell sequencing from CD34-positive CD38-negative (CD34+CD38-) HSCs.

These protocols were employed in order to assess which of these was compatible with cell fixation in terms of sequence quality, yield and ADO rate.

## 2. Results

### 2.1. Cell Fixation Is Necessary for DEPArray Single-Cell Sorting but Can Affect WGA Efficiency

In order to compare different cell fixation methods, ascertain their impact on WGA efficiency and determine which WGA strategy would ensure the best genotyping results by Sanger sequencing, primary CD34+CD38-, HEL or K562 cells were fixed using either Paraformaldehyde (PFA) 2 or 0.5% in PBS 1Xat 4 °C for 15 min 45 min, 2 h or overnight or Methanol (MetOH) 100% 2 h at 4 °C. 

Cells were immunostained and then isolated by means of DEPArray. After DNA isolation from each single cell, five commercially available WGA kits were tested, as detailed in materials and methods section. Amplified DNA was tested for size distribution and for its suitability to be genotyped by Sanger sequencing of the genes most frequently mutated in MPNs (i.e., *JAK2* and *ASXL1*).

**Figure 1:**
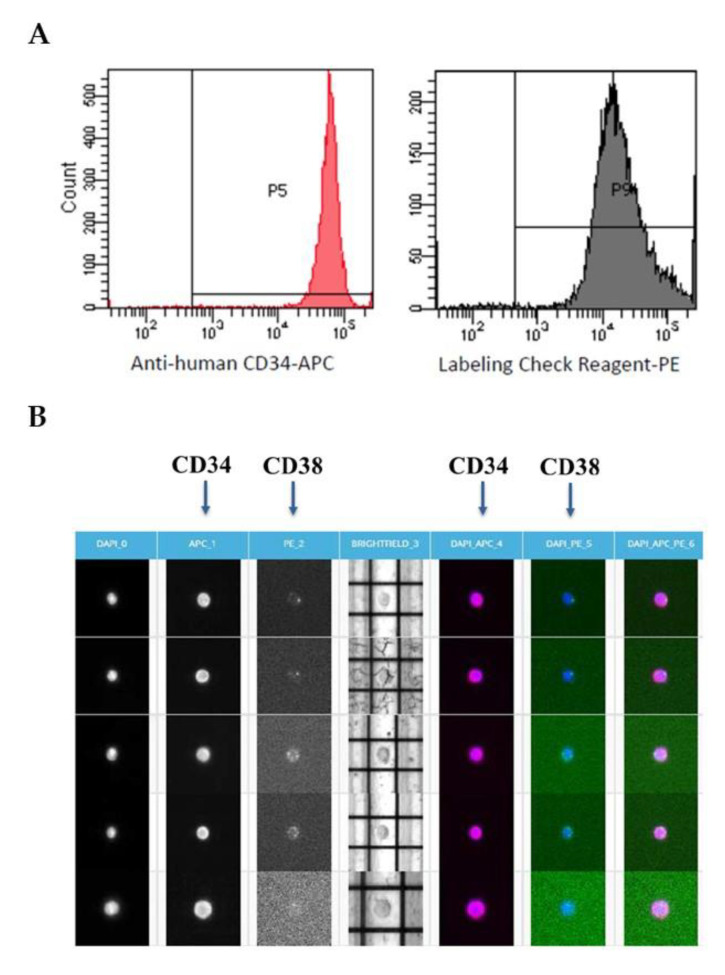
Single-cell sorting using DEPArray. (**A**) Flow cytometry analysis of CD34-positive CD38-negative (CD34+CD38+) cells fixed with 2% PFA overnight. As shown, APC and PE fluorochromes are compatible with PFA. (**B**) Single CD34+/CD38- cells sorted by DEPArray, labeled with anti-CD34-APC and anti-beads-PE. The labeling check reagent-PE identifies CD38 expression. All cells have been subjected to DAPI staining in order to assess their viability.

Cell fixation is obviously a crucial step to preserve fluorescence and assess the purity of the sorted single cells. In order to test the impact of cell fixation on cell viability, immunostaining and sorting efficiency, CD34+CD38- cells were initially fixed with 2% PFA overnight before DEPArray sorting. This fixation strategy proved to be compatible with APC and PE fluorochromes as shown in the flow cytometry analysis of the CD34+CD38+ cell fraction (Figure 1A). Flow cytometry controls of this immunostaining strategy are shown in Figure S1. DEPArray technique is based on the electrokinetic principle of dielectrophoresis, that allows single-cell moving and sorting through the manipulation of a non-uniform electric field. During DEPArray separation, the cell viability was assessed in order to verify the presence of just one viable cell inside the DEPArray cage and to confirm its immunophenotype (i.e., CD34 positivity and CD38 negativity) [12] (Figure 1B). 

After single-cell sorting, cells were subjected to WGA in order to uniformly amplify their genome for downstream sequencing. Three commercially available WGA kits were tested: Ampli1™ WGA Kit, SMARTer PicoPLEX Single Cell WGA kit and REPLI-g Single Cell Kit. 

Ampli1 WGA kit protocol is based on DNA fragmentation through Mse1 restriction enzyme followed by genome amplification, PicoPLEX performs a quasi-random priming pre-amplification step that precedes a uniform isothermal genome amplification, while RepliG protocol is based on MDA by φ29 DNA polymerase.

**Figure 2:**
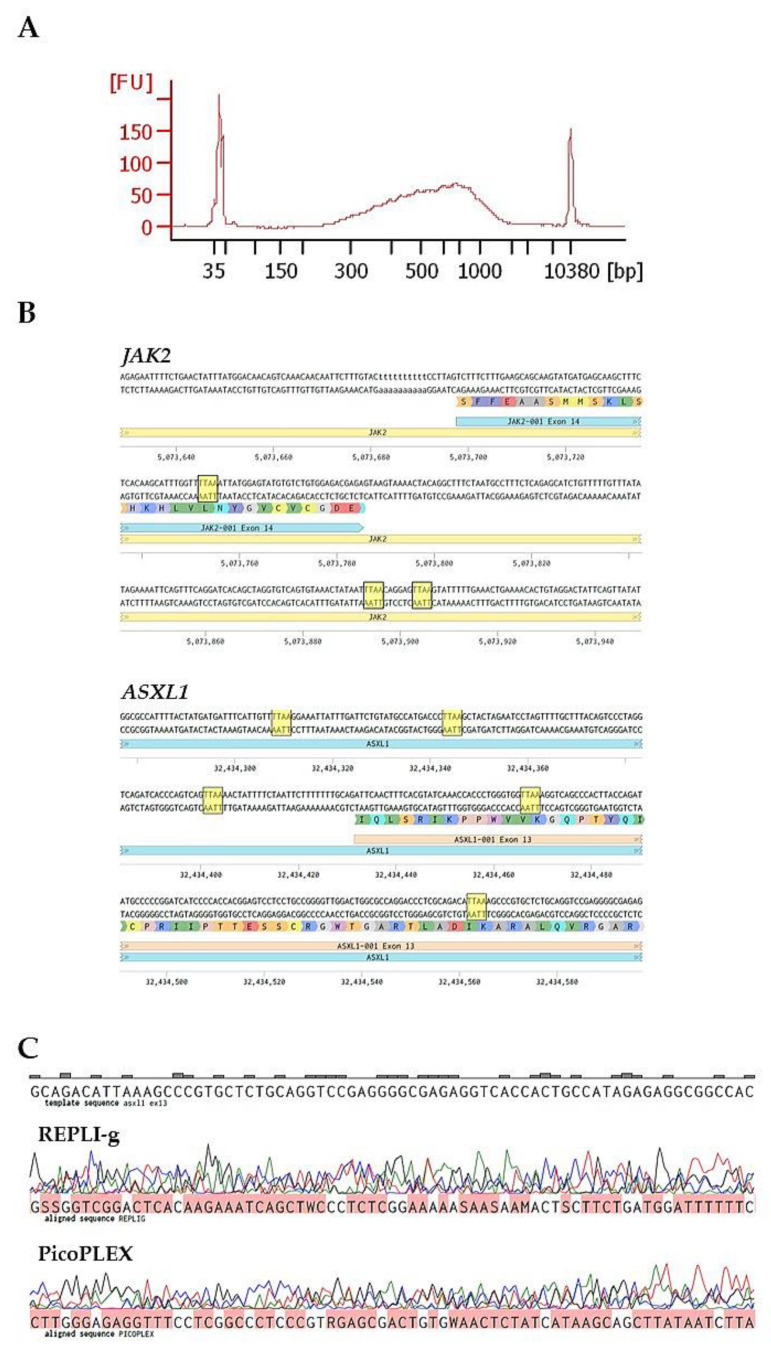
WGA kit’s efficiency on cells fixed overnight with 2% PFA. (**A**) Bioanalyzer profile of amplified DNA, obtained through Ampli1 WGA kit, coming from a single CD34+CD38- cell. (**B**) Cutting frequency of MseI enzyme, used for DNA fragmentation in Ampli1 single-cell WGA kit, in two representative regions of JAK2 and ASXL1 genes. 5’-TTAA-3’ restriction sites are highlighted in yellow. (**C**) Two representative sequences of ASXL1 gene deriving from CD34+CD38- cells (*n* = 20) fixed with PFA 2% overnight. A single-cell genome was amplified with either RepliG or PicoPLEX WGA kit. Both sequences were unreadable highlighting the incompatibility between the kits and the fixation protocol.

The results showed that Ampli1 kit was able to amplify single-cell DNA (Figure 2A), but the Mse1-mediated DNA fragmentation showed important limitations due to its high cutting frequency. In fact, this type of fragmentation often does not allow to design sequencing strategies in regions with known mutations as in the case of *JAK2* and *ASXL1* (Figure 2B). Moreover, in these experimental conditions, Sanger sequencing of *ASXL1* exon 13 coming from PicoPLEX- and RepliG-amplified DNA was inefficient despite the absence of the enzyme digestion step (Figure 2C).

### 2.2. MetOH or Low-Concentration PFA Could Be Alternative Fixation Methods

**Figure 3:**
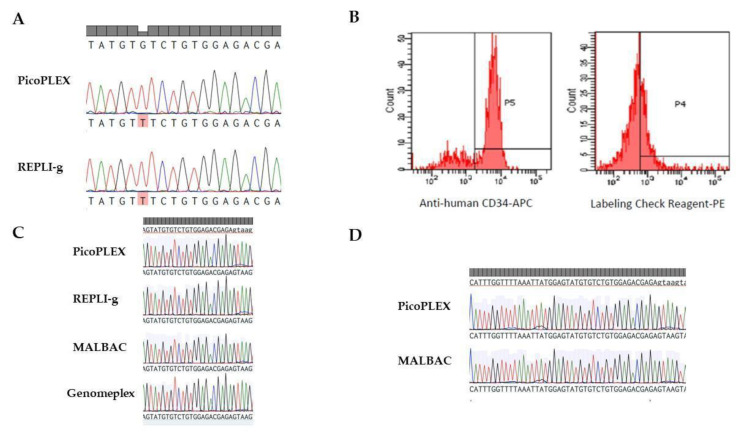
Assays of alternative cells fixation methods. (**A**) Two representative sequences of JAK2 gene coming from unfixed HEL cells whose genome was amplified through either RepliG or PicoPLEX WGA kit (*n* = 20 for each kit). In all cells, the homozygous JAK2 c.1849G>T carried by HEL cells was detected. (**B**) CD34+CD38+ cells fixed with MetOH 100% for 2 h. Flow cytometry analysis shows that MetOH fixation preserves APC fluorochrome but decreases PE signal. The labeling check reagent-PE identifies CD38 expression. (**C**) CD34+CD38+ cells fixed with 100% MetOH for 2 h at 4 °C. Single-cell WGAs were obtained with PicoPlex, RepliG, GenomePlex and MALBAC kits (*n* = 20 for each kit). Electropherograms represent the analysis of a sequence of JAK2 gene. (**D**) CD34+CD38+ cells fixed with 0.5% PFA for 15 min. Single-cell WGAs were obtained with PicoPlex and MALBAC kits (*n* = 20 for each kit). Electropherograms represent the analysis of a sequence of JAK2 gene.

In order to understand if the cell fixation method was responsible for the sequencing failure, unfixed HEL cells were sorted at the single-cell level through limiting dilution and subjected to WGA with PicoPLEX and RepliG kits. Sanger sequencing was subsequently performed on *JAK2* exon 14 of amplified DNA. As expected, WGAs obtained from DNA of unfixed cells resulted in the efficient amplification and sequencing of the target region with Ampli1, PicoPlex and RepliG kits (Figure 3A). 

Taking into account these results, we tried out other fixation strategies in order to find the one(s) compatible with both immunostaining and WGA. First, we performed 100% MetOH fixation for 2 h at 4 °C upon immunostained CD34+CD38+ cells. In addition to previously described WGA methods, we also employed MALBAC and DOP-PCR protocols, i.e., respectively MALBAC and Genomeplex kits. Our results show that, unlike PFA, MetOH fixation is not able to preserve PE staining, therefore proving to be unsuitable for downstream DEPArray or FACS separation (Figure 3B). An alternative immunostaining strategy employing Labeling check reagent-FITC was tested. Our results showed that also FITC fluorescence signal was incompatible with MetOH fixation (Figure S2). Of note, WGAs performed on MetOH fixed cells through PicoPLEX, RepliG, Genomeplex or MALBAC kits resulted in efficient amplification and sequencing of target regions (Figure 3C). MetOH fixation may therefore be compatible with WGAs coming from single cells derived from cell populations that do not require immunostaining.

For this reason, we tested PFA at lower concentrations and for shorter incubation times in order to limit its cross-linking activity upon DNA and histone residues that could hamper optimal genomic amplification. In particular, we tried out 0.5% PFA incubation for 15 min, 45 min or 2 h. In all three conditions, CD34+CD38+ cells immunostaining with APC and PE fluorochromes was preserved, but WGA performed with either RepliG or Genomeplex kit was inefficient (data not shown). On the contrary, low quality WGAs were obtained through PicoPLEX and MALBAC kits in 45 min- and 2 h-fixed cells (data not shown), but showed good efficiency for downstream Sanger sequencing after 15 min of 0.5% PFA fixation (Figure 3D). For this reason, we selected this fixation protocol as optimal for our experimental conditions.

### 2.3. Comparison of ADO Effect in Single-Cell Whole Genome Amplification Kits

After the optimization of the fixation method, we tested the performance of the different WGA kits in terms of yield, efficiency for downstream Sanger sequencing and ADO rate. To this aim, K562 cells were fixed in PFA 0.5% for 15 min and WGA with the five different kits (Ampli1, PicoPlex, RepliG, GenomePlex and MALBAC) after single-cell sorting through DEPArray was performed.

**Figure 4:**
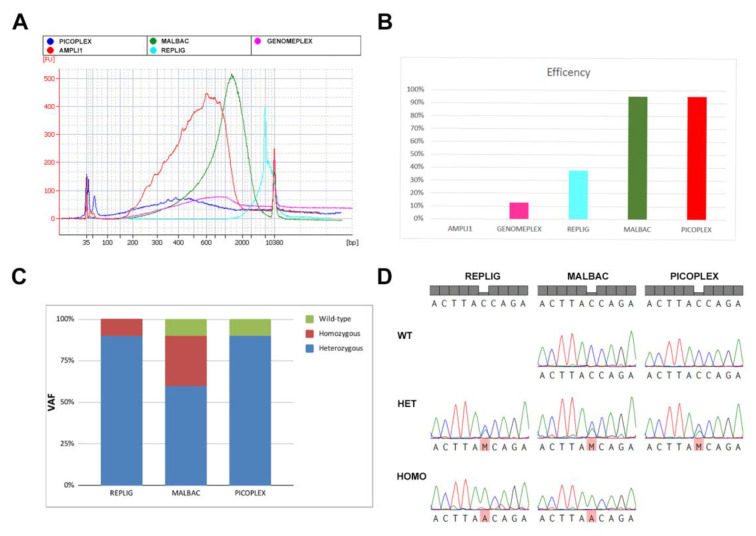
Evaluation of single-cell WGA quality. (**A**) Bioanalyzer profiles of amplified DNA coming from single K562 cells fixed with PFA 0.5% for 15 min. Color code: PicoPlex in red, Ampli1 in blue, Malbac in green, RepliG in light blue and GenomePlex in purple. (**B**) Percentage of sequencing efficiency, performed on a region of the ASXL1 gene, obtained with single-cell WGA kits (*n* = 20 cells for each kit). (**C**) Histogram representing the rate of ADO effect obtained with the analysis of the heterozygous variant of ASXL1 gene (c.1773C>G) carried by K562 cell line (*n* = 20 cells for each kit). (**D**) Representative peaks of homozygous, heterozygous and wild-type alleles identified using Sanger sequencing. Sequences were obtained by different single-cell WGA kits (*n* = 20 cells for each kit) and were related to the heterozygous variant of ASXL1 gene (c.1773C>G) carried by K562 cell line.

Bioanalyzer run performed on different WGA products showed that PicoPLEX and MALBAC amplification products were characterized by highest yield and the larger size distribution. On the contrary, Ampli1 and Genomeplex had a markedly lower yield. Moreover, Ampli1 fragments were shorter than the ones produced by the other kits, highlighting once more the limitations of this WGA protocol. RepliG fragments were longer than the ones generated by other kits, due to the high processivity of φ29 DNA polymerase (Figure 4A). A statistical analysis of the WGA yield and the average fragment length coming from the five kits can be found in Figure S3.

In order to assess the WGAs’ suitability for downstream Sanger sequencing, we sequenced a portion of *ASXL1* exon 13 in K562 WGAs coming from the different kits. In WGA samples performed with Ampli1 kit, the region of interest results unamplified due to Mse1 digestion (Figure 2B). Despite the good yield, RepliG derived WGA showed to be quite inefficient for Sanger sequencing of this region (37.50% of sequencing efficiency). On the contrary, PicoPLEX and MALBAC showed both a good yield and a high Sanger sequencing efficiency (95%) (Figure 4B). 

To evaluate the ADO rate resulting from the kits allowing a good sequencing efficiency (RepliG, PicoPLEX and MALBAC), we performed Sanger sequencing of a heterozygous variant of *ASXL1* gene (c.1773C>G) carried by K562 cells. Our results show that both RepliG and PicoPLEX had a low ADO rate; on the contrary, MALBAC kit produced a higher ADO rate since it detected either wild-type or homozygous cells as shown by histograms and representative electropherograms in Figure 4C,D.

As a whole, these analyses showed that PicoPLEX is the only kit among those tested featuring the highest amplification yield, the highest suitability for downstream sequencing and the lowest ADO rate (Table 1).

## 3. Discussion

Single-cell WGA is an essential step in analyzing the mutational asset of single cells due to the low amount of genomic material (<10 pg) [13]. In recent years, this approach has been taken by many molecular oncology laboratories, thanks to its capability to draw the complex picture that characterizes polyclonal and/or multi-step carcinogenesis.

For this reason, single-cell analysis has proved to be very useful for the study of cellular subpopulation in many solid human tumors, such as breast [1], kidney [2], colon [3] and prostate [4] cancers.

In this work, we aimed at optimizing a workflow for cell immunostaining, fixation and WGA dedicated to the single-cell genomic analysis of the hematopoietic CD34+CD38- stem compartment, in which originate hematological malignancies such as Myeloproliferative Neoplasms.

In our experimental design, cell fixation was performed in order to preserve cell immunostaining and allow an efficient single cell sorting through DEPArray technology [12].

However, the use of fixative agents such as PFA or MetOH can impair the uniform genome amplification due to their cross-linking effect between DNA and histone residues. Our results showed that a strong PFA fixation is compatible with Ampli1 WGA kit, but this WGA strategy is strongly limited by Mse1 restriction enzyme employment. This enzyme is a frequent cutter inside the human genome, as exemplified by the Mse1 restriction sites in two regions of JAK2 and ASXL1 genes (Figure 2). These genes are frequently mutated in MPNs [14,15]. In particular, Mse1 restriction sites are found in close proximity to the exon 14 site in which originates the JAK2V617F mutation, making the variant discrimination very difficult.

On the other hand, a strong PFA fixation is not compatible with WGA kits that skip the enzymatic fragmentation, such as PicoPlex and RepliG (Figure 2B). The link between WGA efficiency and cell fixation was confirmed by experiments performed by these kits in unfixed HEL single cells. Sanger sequencing of selected regions performed on these WGA preparations was successful, confirming the deleterious effect of strong PFA cell fixation on downstream analyses (Figure 3A).

The use of MetOH as an alternative cell fixative resulted in efficient WGA and Sanger sequencing with most of the commercially available WGA kits (PicoPlex, RepliG, Genomeplex and MALBAC) (Figure 3C). However, MetOH fixation strongly hindered the preservation of cell immunostaining, in particular PE fluorescence, proving to be unsuitable for single-cell sorting with DEPArray system (Figure 3B). Unlike PFA, MetOH fixative action is based on cell permeabilization, causing strong cell dehydration and proteins precipitation, but showing a higher preservation of nucleic acid structure thanks to the absence of the cross-linking effect peculiar of aldehyde-based fixatives [16]. For this reason, we suggest the use of this fixation strategy for single-cell sorting of cell populations which have not been subjected to previous immunostaining. 

CD34+CD38- cell fixation with lower PFA concentrations performed for shorter time in order to mitigate its cross-linking effect, resulted in the efficient genome amplification with PicoPlex and MALBAC kits (both based on the MALBAC amplification strategy) (Figure 3D), but was unsuccessful with RepliG and GenomePlex kits (based on MDA and DOP-PCR approaches, respectively).

Using 0.5% PFA for 15’ as the optimal fixation protocol for CD34+CD38- cells, the five WGA kits were tested in these experimental conditions on single K562 cells in order to assess DNA yield, ADO rate and efficiency of downstream Sanger sequencing (Figure 4).

WGA yields and fragment size distribution reflected the molecular approaches followed by the different kits. PicoPlex and MALBAC showed the higher yield and the larger fragment size distribution, due to their quasi-random priming strategy and efficient and uniform amplification of the hairpin molecules generated during the pre-amplification step. On the other hand, Ampli1 WGA kit was characterized by a markedly lower yield and shorter fragment size (Figure S3), confirming once again the limitations associated with Mse1 digestion. RepliG protocol, based on the highly processive φ29 DNA polymerase, generated the longest WGA fragments, but failed to provide DNA suitable for Sanger sequencing. Conversely, MALBAC-based approaches showed a high sequencing efficiency of the region of interest, suggesting that the looping-based amplification allows a uniform coverage of genomic DNA.

Finally, we proceeded with ADO rate assessment, which is a fundamental parameter for the evaluation of a correct single-cell genotyping in terms of variant zygosity. Our data demonstrate that RepliG and PicoPlex kits are able to detect heterozygous mutations with a higher accuracy than MALBAC, showing a higher rate of false positives (homozygous mutants) and negatives (wild-type) (Figure 4D).

As a whole, our study demonstrated that a mild PFA fixation (0.5% PFA for 15 min) is compatible with CD34+CD38- cell immunostaining, DEPArray sorting and single-cell WGA. In conclusion, the PicoPlex WGA kit, adopting MALBAC strategy, was found to be the better performing kit in terms of WGA yield, fragment size, sequencing efficiency and ADO rate. 

In order to perform single-cell experiments, it is important to pay attention to robustness and reproducibility of the protocol, since the biological material used in these procedures is often precious or not recoverable. It is important to highlight how the experimental procedure described in this work was aimed at the validation, by means of Sanger sequencing, of genomic variants previously characterized by Next Generation Sequencing (NGS) analysis in bulk. NGS single-cell genomics strategies have recently been developed either following WGA protocols or through the employment of a droplet-based targeted strategy (i.e., Tapestri platform [17]). The technique described in this work, unlike NGS, is suitable for a larger spectrum of diseases, since the sequencing panels designed for droplet-based techniques, as of today are available only for a restricted number of neoplasms (e.g., panels for Sarcomas, Mesothelioma and Neuroblastoma are not yet available). Moreover, hematological malignancies tend to evolve into one another (i.e., MPNs could progress to Acute Myeloid Leukemia [18]), and therefore the study of a single patient would require the design of a custom panel able to join together two or more pre-designed panels.

This workflow could therefore be adopted in order to understand the clonal complexity and evolution of solid and hematological malignancies sustained by multiple clones whose prevalence is modulated during disease progression. Moreover, this novel single-cell genomics approach is a powerful instrument to gain insights into allele distribution in a cell population, understand cells’ zygosity and highlight the presence of rare genetic variants.

Noteworthy, in clinical management, the early detection of malignant clones through single-cell genomic analysis could be useful to allow a better prognostic assessment of the patients and the selection of personalized therapies. 

## 4. Materials and Methods 

### 4.1. Ethics Statement

Human CD34+38- HSCs were purified upon donor’s informed written consent from umbilical Cord Blood samples, collected after normal deliveries, according to the institutional guidelines for discarded material (Clearance of Ethical Commitee for Human experimentation of Florence: Comitato Etico Area Vasta dell’Azienda Ospedaliero-Universitaria Careggi, approval date: 22 April 2011, approval file number # 2011/ 0014777). 

### 4.2. CD34+38- Cell Purification, Fixation and Immunostaining

Cord blood CD34+38- were purified by means of CD34+CD38- Cell Isolation Kit, human (Miltenyi Biotec; Bergisch Gladbach, Germany). Cell purity was assessed by means of flow cytometry after immunostaining with anti-human CD34-APC (1:50) and Labeling Check Reagent-PE (1:33), Labeling Check Reagent-FITC (1:33) or antibodies (Miltenyi Biotec). The labeling check reagent-PE identify CD38 expression by binding the beads bond to the CD38 antigen. Immunostained cells were then fixed with either Paraformaldehyde (PFA) 2% or 0.5% in PBS1× at 4 °C for 15 min, 45 min, 2 h or overnight or MetOH 100% 2 h at 4 °C and washed with PBS1× before being resuspended in autoMACS Running Buffer (Miltenyi Biotec).

### 4.3. CD34+38- Cell Sorting

Immunostained CD34+38- cells were then subjected to single-cell sorting by means of DEPArray™ System (Menarini Silicon Biosystems, Castel Maggiore, BO, Italy) [12]. The sorted population was made up of CD34+CD38- cells with a purity of 100%. All cells have been subjected to DAPI staining in order to assess viability. Each single cell was resuspended in 1 μL of 1XPBS.

### 4.4. Cell Lines Culture and Sorting

In order to assess the ADO rate of each WGA kit, the evaluation of known Single Nucleotide Variants was performed by means of Sanger sequencing on HEL and K562 cell lines, since they carry known genomic variants either in homozygous or heterozygous state. HEL and K562 cell lines were obtained from the ATCC and cultured in RPMI medium (Euroclone S.p.A, Pero, MI, Italy), supplemented with 10% heat-inactivated FBS (Sigma-Aldrich, St. Louis, MO, USA) and 1 mmol/L l-Glutamine (Euroclone) [19]. HEL cells (JAK2V617F homozygous mutant) were sorted as single cells in 1 μL of 1×PBS by means of limiting dilution [20]. K562 cells (ASXL1 c.1773C>G heterozygous mutant) were fixed with PFA 0.5% in PBS1× at 4 °C for 15’ and sorted as single cells through DEPArray™ Technology (Menarini Silicon Biosystems). 

### 4.5. Single-Cell Whole Genome Amplification

Whole Genome Amplification was performed on 20 single CD34+CD38-, HEL or K562 cells by means of SMARTer PicoPLEX Single Cell WGA kit (Takara Bio Inc, Kusatsu, Japan), REPLI-g Single Cell Kit (Qiagen, Hilden, Germany), Ampli1™ WGA Kit (Menarini Silicon Biosystems), MALBAC Single Cell WGA Kit (Yikon Genomics, Beijing, China) or GenomePlex^®^ Single Cell Whole Genome Amplification Kit (Sigma-Aldrich) following manufacturer’s protocol. For each WGA experiment, a genomic DNA sample and 1uL of Low Tris-EDTA (TE) buffer (Thermo Fisher Scientific, Waltham, MA, USA) were included as positive and negative controls, respectively. WGA product was then purified by means of AMPure XP (Beckman Coulter, Brea, CA, USA) immunomagnetic beads and eluted in 20 μL of Low TE buffer. Quality control on WGA yield and amplicons’ size was performed through Bioanalyzer High Sensitivity DNA Analysis (Agilent Technologies, Santa Clara, CA, USA).

### 4.6. Sanger Sequencing

PCR and sequencing analysis were performed by using BigDye^®^ Direct Cycle Sequencing Kit (Applied Biosystem^®^, Foster City, CA, USA) following the manufacturer’s protocol. Primers used for PCR reactions (Thermo Fisher Scientific and Integrated DNA Technologies, Coralville, IA) were synthesized with the addition of the M13 tail (M13 FW: 5′ TGTAAAACGACGGCCAGT 3′; M13 RV: 5′ CAGGAAACAGCTATGACC 3′). Sequencing products were purified by ethanol/EDTA precipitation. Sequencing was performed by capillary electrophoresis on 3130xl Genetic Analyzer (Applied Biosystems^®^).

## Figures and Tables

**Table 1:** Table summarizing the characteristics and the compatibility of WGA kits.

WGA Method	Compatible with PFA Fixation (High Concentration and Long Time)	Compatible with PFA Fixation (Low Concentration and Short Time)	Compatible with MetOH Fixation	Efficiency (%)	ADO Effect	Sequence Quality
Ampli1	Yes	Yes	Yes	0	-	-
Genomeplex	No	No	Yes	12.5	-	-
REPLI-g	No	No	Yes	37.5	Low	Low
PicoPLEX	No	Yes	Yes	95	Low	High
MALBAC	No	Yes	Yes	95	High	Medium

## Supplementary Materials

Supplementary materials can be found at https://www.mdpi.com/1422-0067/21/19/7366/s1.

## Abbreviations

HSC	Hematopoietic Stem Cell
MPN	Myeloproliferative Neoplasm
scWGA	Single cell Whole Genome Amplification
FACS	Fluorescence-Activated Cell Sorting
WGA	Whole Genome Amplification
MDA	Multiple Displacement Amplification
DOP-PCR	Degenerative Oligonucleotide PCR
MALBAC	Multiple Annealing and Looping-Based Amplification
ADO	Allelic Dropout
PFA	Paraformaldehyde
MetOH	Methanol
NGS	Next Generation Sequencing
