# Supplementary material for: Genomic Analysis of Hematopoietic Stem Cell at the Single-Cell Level: Optimization of Cell Fixation and Whole Genome Amplification (WGA) Protocol
Source: Int J Mol Sci. 2020 Oct 6;21(19):7366. doi: 10.3390/ijms21197366 (PMC7582552; doi:10.3390/ijms21197366)
Supplement: Supplementary file 1 [file ijms-21-07366-s001.pdf]

"Genomic Analysis of hematopoietic stem cell at the Single-Cell Level: optimization of cell fixation and Whole Genome Amplification (WGA) protocol" By Carretta and Mallia et al,

SUPPLEMENTARY MATERIAL

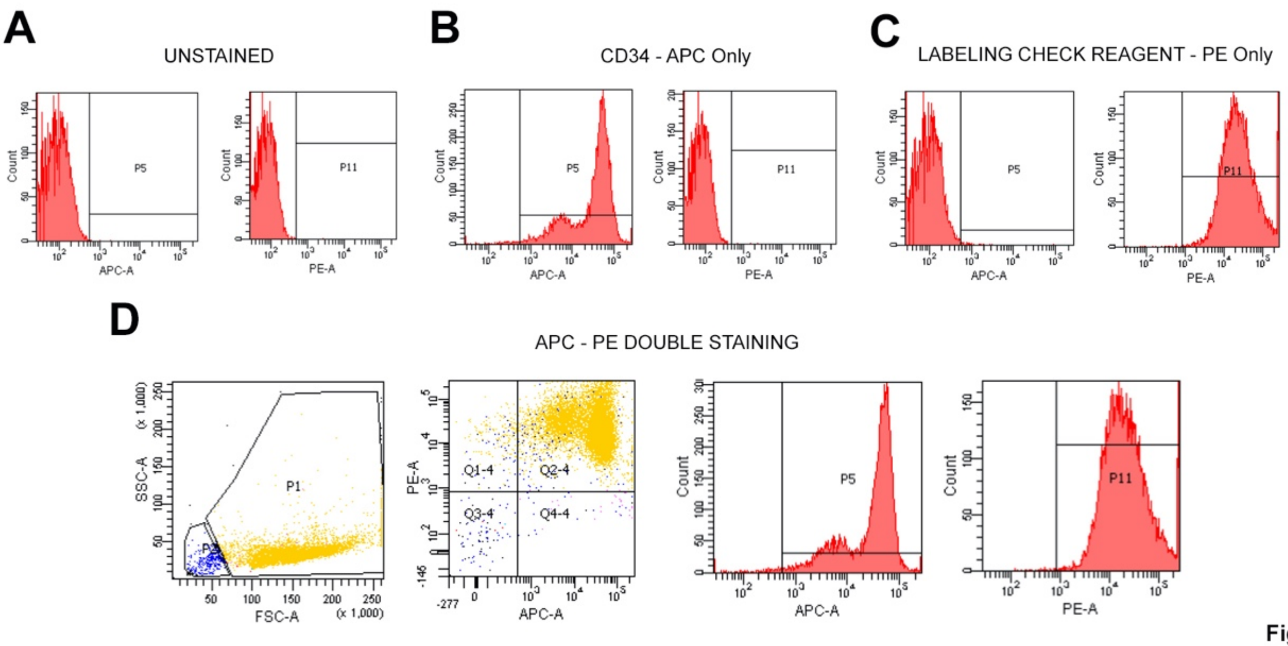

Figure S1

Figure S1. Cytofluorimetric controls referring to CD34-APC/Labeling check reagent-PE immunostaining. **(A)** Unstained control of unfixed CD34+CD38+ cells. **(B)** CD34-APC single staining performed on unfixed CD34+/CD38+ cells. **(C)** Labeling check reagent-PE single staining performed on unfixed CD34+/CD38+ cells. **(D)** CD34-APC/Labeling check reagent-PE double staining performed on unfixed CD34+CD38+ cells.

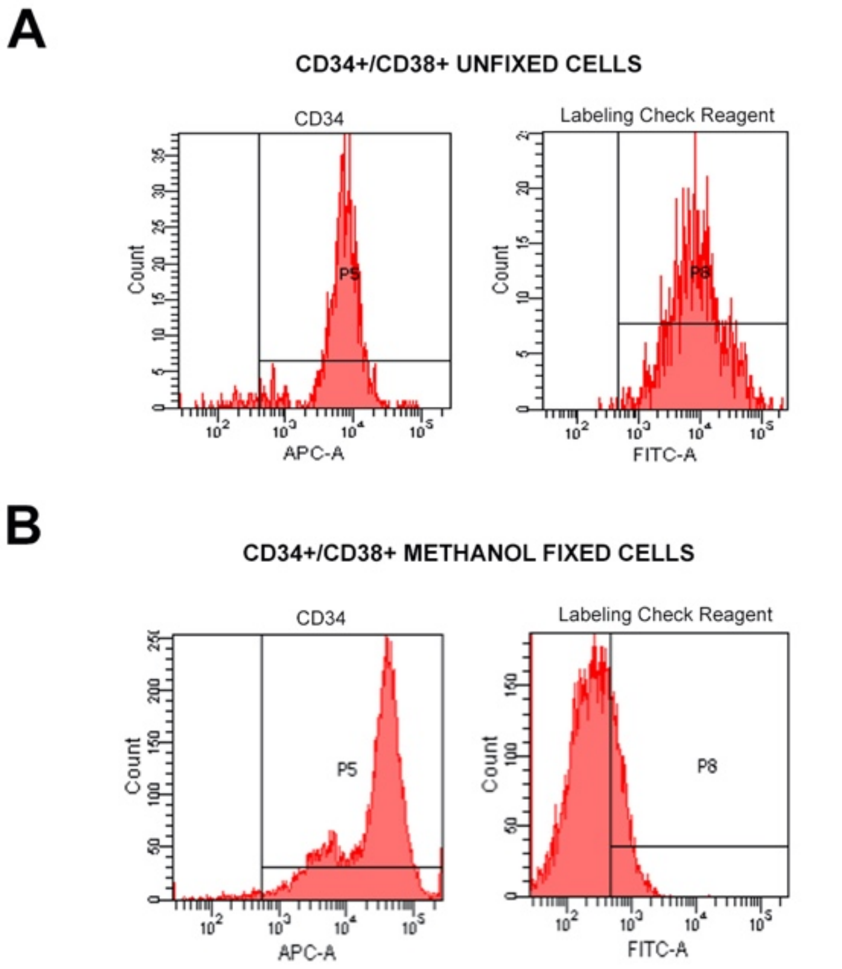

Figure S2

Figure S2. Cytofluorimetric analysis of CD34-APC/Labeling check reagent-FITC immunostaining. **(A)** Double staining performed on unfixed CD34+/CD38+ cells. **(B)** Double staining performed on 100% MetOH fixed CD34+/CD38+ cells.

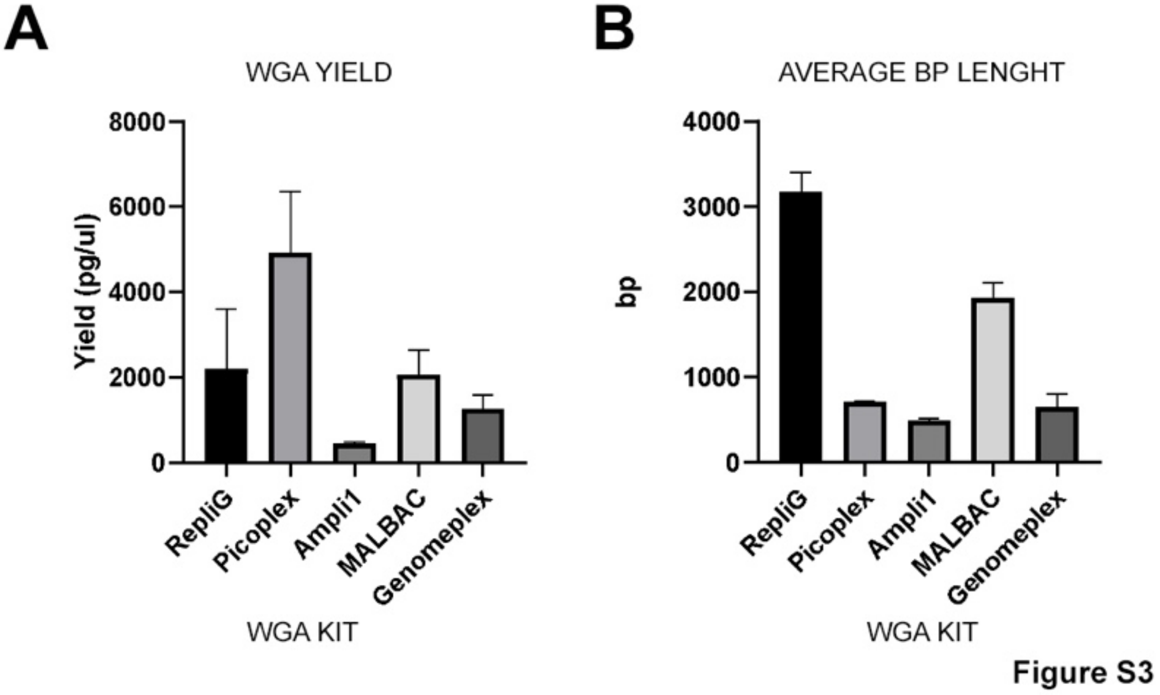

Figure S3. Comparison of WGA yield and average fragment length of the different WGA kits. **(A)** Analysis of the WGA yield of PFA 0.5% 15' fixed K562 cells subjected to WGA with five different WGA kits (n=5 for each kit). **(B)** Analysis of the average length of the genomic fragments coming from PFA 0.5% 15' fixed K562 cells subjected to WGA with five different WGA kits (n=5 for each kit). Data are shown as mean ± SEM.
